# 2,5-Dihydroxyphenylethanone: an anti-melanogenic bioactive compound isolated from *Ganoderma cochlear*

**DOI:** 10.1080/14756366.2025.2495364

**Published:** 2025-04-29

**Authors:** Meng Ning, Xiao-Cui Liu, Min He, Xing-Rong Peng, Ming-Hua Qiu

**Affiliations:** a State Key Laboratory of Phytochemistry and Natural Medicines, Kunming Institute of Botany, Chinese Academy of Sciences, Kunming, China; b University of Chinese Academy of Sciences, Beijing, China

**Keywords:** 2,5-dihydroxyacetophenone, anti-melanogenesis, chemical synthesis, zebrafish, *Ganoderma cochlear*

## Abstract

2,5-dihydroxyacetophenone, a natural product from the fruiting bodies of *Ganoderma cochlear*, can effectively and safely inhibit the production of melanin in zebrafish model. To achieve analogues with more significant inhibition, 9 analogs were synthesised and 13 analogues were purchased commercially. Among them, 14 compounds can inhibit melanin production, of which 5 compounds displayed the most significant inhibitory effects, with inhibitory rates of more than 80%, compared to positive control SymWhite^®^377 (phenylethyl resorcinol). This study elucidated the melanin-inhibitory effects of 2,5-dihydroxyacetophenone and its analogs, providing a theoretical foundation for their potential applications in anti-melanogenic reagents.

## Graphical Abstract

**Figure:**
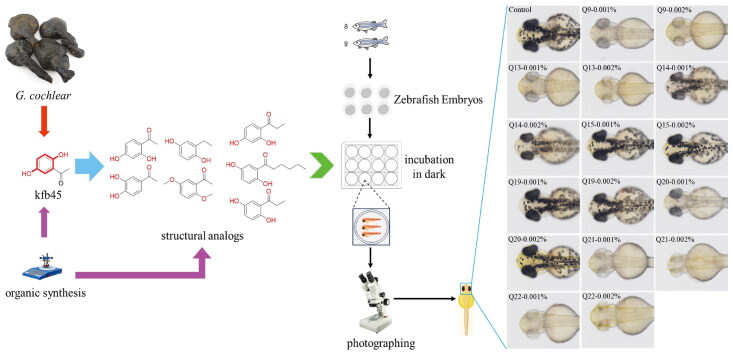

## Introduction

Melanin is a pigment that exists on the surface of the skin and affects the colour of the skin in organisms. It can protect the skin from various injuries under ultraviolet light^1^^,^^2^. However, if the content of melanin increases, there may be various types of scars on the skin surface caused by excessive pigmentation^3^^,^^4^. During the formation of melanin, tyrosinase catalyses L-tyrosine to L-3,4-dihydroxyphenylalanine, which biochemical reaction is the rate-limiting step in melanin synthesis. L-3,4-dihydroxyphenylalanine is further oxidised to *o*-dopaquinone, and then the subsequent reaction is carried out. Therefore, the purpose of reducing melanin production can be achieved by inhibiting the activity of tyrosinase^5–8^. Compared with chemically synthesised reagents, natural-derived compounds are always mild and safe. It is of great research significance to find new compounds that inhibit tyrosinase activity and/or various targets in the melanin synthesis pathway from natural sources^9^.

To date, tyrosinase inhibitors with diverse structures have been developed from various natural, semi-synthetic, and synthetic compounds aiming to the key target of melanin synthesis, tyrosinase^10^. Currently, various types of compounds with reported structures, such as ascorbic acid, heterocyclic derivatives, aromatic derivatives, tyrosinase-PROTAC hybrids, amino acid-based derivatives^10^, arbutin, kojic acid^11^^,^^12^, thiazolyl resorcinol^13^, hydroquinone^14^, thiourea compounds^15^, thiazolidine derivatives^16^, aryl pyrazole^17^, sulphonamide derivatives^18^, have shown good inhibition on tyrosinase.

Zebrafish have been validated as a reliable vertebrate model for screening melanogenesis inhibitors^19–21^. Evolutionarily, zebrafish pigment cells are highly conserved among vertebrates^22^. On the other hand, there is a similarity between the human genome and the zebrafish genome, as well as there are many similarities between human skin and zebrafish skin, indicating that zebrafish is suitable as model organisms for studying the inhibition of melanogenesis^23^. In addition, during the early development of zebrafish, the body is transparent, and at 24 h after embryonic development, melanin begins to form on the retinal epithelium of the zebrafish. Pigment cells originate from a group of cells differentiated from the dorsal ectoderm, known as neural crest cells, which proliferate, migrate, and differentiate into melanoblasts^24–26^. Interrupting the process of melanin formation may inhibit the formation of melanin^27^. Therefore, the whiteness of zebrafish skin can be evaluated the whitening efficacy of the samples^28^^,^^29^.

In the previous study, a natural source of 2,5-dihydroxyacetophenone was found from the wild *Ganoderma cochlear*. Considering the compound is structurally similar to hydroquinone and is likely to have a whitening and freckle-removing effect, it was investigated in terms of inhibiting melanogenesis. Furthermore, structural analogs of 2,5-dihydroxyacetophenone were systematically investigated for their melanogenesis-inhibitory properties. Through comparative analysis, compounds exhibiting significant inhibitory effects were identified. This study was designed to provide validated research foundations for the development of effective melanin synthesis inhibitors based on the 2,5-dihydroxyacetophenone pharmacophore.

## Materials and methods

### Plant material

*Ganoderma cochlear* belongs to the kingdom Fungi, division Basidiomycota, class Agaricomycetes (formerly Homobasidiomycetes), order Polyporales (replacing Aphyllophorales), and family Ganodermataceae^30^. The fruiting body of *Ganoderma cochlear* was purchased from the Chinese herbal medicine market in Juhua Village, Yunnan Province in August 2013. The specimen was authenticated by Professor Peigui Liu of the Kunming Institute of Botany, Chinese Academy of Sciences (voucher number: KIB No. 13081001), and is deposited in the Phytochemistry and Plant Resources Research Group at the State Key Laboratory of Phytochemistry and Natural Medicines, Kunming Institute of Botany, Chinese Academy of Sciences, Xing-Rong Peng is the person in charge (E-mail: pengxingrong@mail.kib.ac.cn).

### General experimental procedures

All reagents, solvents and materials are commercially available, reagents and solvents do not require further purification. Tyrosinase (from mushroom) was purchased from Zhejiang Maifei Biotechnology Co., Ltd., No.: T3824-25KU, brand: SIGMA. The reaction process was monitored by thin layer chromatography (TLC) under 254 nm ultraviolet light, and the compounds were purified by 200–300 mesh normal phase silica gel column chromatography. The ^1^H NMR spectra (400, 600 MHz) and ^13^C NMR spectra (100, 150 MHz) of all compounds were recorded in chloroform-*d*_1_ and DMSO-*d*_6_ solutions (Bruker, Germany). High resolution mass spectrometry (HRMS) was performed on an Agilent 6540 series quadrupole time-of-flight (Q-TOF) mass spectrometer (Agilent, Germany). All compounds used in this study were obtained either through chemical synthesis or commercial procurement, commercially available compounds: phenethyl resorcinol, kojic acid, Q1, Q2, Q3, Q4, Q5, Q6, Q7, Q9, Q10, Q11, Q12, Q17 and Q18. The reported yields refer to purified products isolated by column chromatography with calculated mass.

### Extraction and isolation

The fruiting bodies of *Ganoderma cochlear* (68 kg) were pulverised and subjected to triple ethanol reflux extraction. The combined extracts were concentrated to yield a crude extract (6 kg). The extract was then suspended in water and partitioned with ethyl acetate. The ethyl acetate fraction (3 kg) was decolourised using macroporous adsorption resin (D101, MeOH-H_2_O) and eluted with a methanol-water gradient. Four fractions (I–IV) were collected at 50%, 70%, 90%, and 100% methanol-water (*v*/*v)*. From Fraction II (480 g), a 50 g aliquot was further purified by reversed-phase silica gel column chromatography, eluting with a 40% → 70% (*v*/*v*) methanol-water gradient. The eluate was concentrated every 500 ml and monitored by TLC for pooling. A major fraction rich in A-ring-seco nortriterpenoids was obtained during the 60% → 70% methanol-water elution. After removing these dominant components, the remaining material was subdivided into eight subfractions (II-1 → II-8). Subfraction II-5, containing dihydroxyacetophenone derivatives, was further fractionated by Sephadex LH-20 column chromatography (mobile phase: methanol) to yield four portions (II-5–1 → II-5–4). Among these, II-5–3 was purified by normal-phase silica gel column chromatography using a petroleum ether-acetone gradient (50:1 → 2:1, *v*/*v*), affording 2,5-dihydroxyacetophenone (56 mg, purity ≥ 99%)^31^. The NMR and mass spectrometry data were in full agreement with published structural assignments^32^, the residual organic solvents were below detectable limits.

### Chemistry

#### General synthesis methods of Q13, Q21 and Q22

In general, ZnCl_2_: 2RCOOH (R = CH_3_, CH_2_CH_3_, (CH_2_)_4_CH_3_) is prepared by stirring ZnCl_2_ (20 mmol) and RCOOH (40 mmol) at 60 °C until ZnCl_2_ is completely dissolved in carboxylic acid, and finally a clear and highly viscous liquid is obtained^33^.


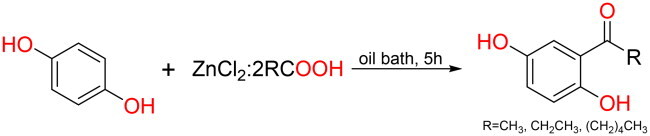


In a 10 ml round-bottom flask, hydroquinone (110 mg) and ZnCl_2_·2RCOOH (R = CH_3_, CH_2_CH_3_, (CH_2_)_4_CH_3_; 400 μL) were added. The mixture was magnetically stirred in an oil bath at 130–170 °C for 5 h, with reaction progress monitored by TLC. After cooling to room temperature, the reaction was quenched with 2 ml of 10% NaHCO_3_ solution to neutralise residual acids. The product was extracted with ethyl acetate (3 × 5 ml), and the combined organic layers were concentrated under reduced pressure. Purification was performed by normal-phase silica gel column chromatography using ethyl acetate/petroleum ether gradients.

Q13: light yellow powder, yield: 39.75%; HR-EIS [M − H]^−^ *m*/*z*: 165.0820; ^1^H NMR (600 MHz, DMSO-*d*_6_) *δ* (ppm): 11.33 (1H, s, 2′-OH), 9.18 (1H, s, 5′-OH), 7.18 (1H, d, *J* = 2.9 Hz, 6′-H), 6.97 (1H, dd, *J* = 8.8, 3.0 Hz, 4′-H), 6.79 (1H, d, *J* = 8.8 Hz, 3′-H), 3.02 (2H, q, *J* = 7.2 Hz, CH_2_), 1.06 (3H, t, *J* = 7.2 Hz, CH_3_); ^13^C NMR (150 MHz, DMSO-*d*_6_) δ(ppm): 206.30 (C = O), 153.58 (2′-C–OH), 149.36 (5′-C–OH), 124.11 (4′-CH), 119.87 (3′-CH), 118.36 (1′-C), 114.63 (6′-CH), 32.03 (CH_2_), 8.06 (CH_3_). The NMR and mass spectrometry data were in agreement with published structural assignments^34^.

Q21: light yellow powder, yield: 39.47%; HR-EIS [M − H]^−^ *m*/*z*: 151.0409；^1^H NMR (600 MHz, DMSO-*d*_6_) *δ* (ppm): 11.39 (1H, s, 2′-OH), 9.43 (1H, s, 5′-OH), 7.14 (1H, d, *J* = 3.0 Hz, 6′-H), 6.97 (1H, dd, *J* = 8.9, 3.0 Hz, 4′-H), 6.77 (1H, d, *J* = 8.9 Hz, 3′-H), 2.53 (3H, s, CH_3_)；^13^C NMR (150 MHz, DMSO-*d*_6_) *δ* (ppm): 204.89 (C = O), 154.33 (2′-C–OH), 149.68 (5′-C–OH), 125.14 (4′-CH), 120.48 (1′-C), 118.86 (3′-CH), 115.85 (6′-CH), 27.94 (CH_3_). The NMR and mass spectrometry data were in agreement with published structural assignments^32^.

Q22: brown oil, yield: 39.90%; HR-EIS [M − H]^−^ *m*/*z*: 207.1497；^1^H NMR (600 MHz, DMSO-*d*_6_) *δ* (ppm): 11.34 (1H, s, 2′-OH), 9.17 (1H, s, 5′-OH), 7.18 (1H, d, *J* = 3.0 Hz, 6′-H), 6.97 (1H, dd, *J* = 8.8, 3.0 Hz, 4′-H), 6.78 (1H, d, *J* = 8.9 Hz, 3′-H), 2.97 (2H, t, *J* = 7.3 Hz, 2-CH_2_), 1.59 (2H, p, *J* = 7.3 Hz, 3-CH_2_),1.34–1.25 (4H, m, 4,5-2 × CH_2_), 0.86 (3H, t, *J* = 7.0 Hz, 6-CH_3_)；^13^C NMR (150 MHz, DMSO-*d*_6_) *δ* (ppm): 206.04 (C = O), 153.68 (2′-C–OH), 149.36 (5′-C–OH), 124.18 (4′-CH), 119.98 (1′-C), 118.39 (3′-CH), 114.72 (6′-CH), 38.64 (2-CH_2_), 30.83 (4-CH_2_), 23.59 (3-CH_2_), 22.00 (5-CH_2_), 13.87 (6-CH_3_).

#### The general synthesis method of Q14 and Q19


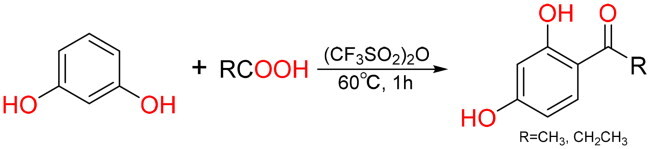


In a 10 ml round-bottom flask, acetic acid (1 mmol, 57 μL) or propionic acid (1 mmol, 75 μL) and trifluoromethanesulfonic anhydride (1.1 mmol, 180 μL) were added, mixed and stirred at room temperature for 1 min, then heated to 60 °C, resorcinol (1 mmol, 110 mg) was added, stirred at 60 °C for 1 h and the reaction was detected by TLC^35^. After the reaction, 3 ml 10% NaHCO_3_ was slowly added, ethyl acetate was extracted multiple times, the ethyl acetate extract was concentrated under reduced pressure, and the product was purified by normal-phase silica gel column chromatography with ethyl acetate and petroleum ether.

Q14: white powder, yield: 97.49%; HR-EIS [M − H]^−^ *m*/*z*: 165.0726; ^1^H NMR (600 MHz, DMSO-*d*_6_) *δ* (ppm): 12.63 (1H, s, 2′-OH), 10.60 (1H, s, 4′-OH), 7.76 (1H, d, *J* = 8.8 Hz, 6′-H), 6.36 (1H, dd, *J* = 8.8, 2.5 Hz, 5′-H), 6.23 (1H, d, *J* = 2.5 Hz, 3′-H), 2.96 (2H, q, *J* = 7.3 Hz, CH_2_), 1.06 (3H, t, *J* = 7.3 Hz, CH_3_); ^13^C NMR (150 MHz, DMSO-*d*_6_) *δ* (ppm): 205.24 (C = O), 164.64 (4′-C–OH), 164.16 (2′-C–OH), 132.82 (6′-CH), 112.30 (1′-C), 108.10 (5′-CH), 102.41 (3′-CH), 30.68 (CH_2_), 8.43 (CH_3_). The NMR and mass spectrometry data were in agreement with published structural assignments^36^.

Q19: white powder, yield: 44.74%; HR-EIS [M − H]^−^ *m*/*z*: 151.0374; ^1^H NMR (600 MHz, DMSO-*d*_6_) *δ* (ppm): 12.60 (1H, s, 2′-OH), 10.63 (1H, s, 4′-OH), 7.74 (1H, d, *J* = 8.8 Hz, 6′-H), 6.36 (1H, dd, *J* = 8.8, 2.5 Hz, 5′-H), 6.23 (1H, d, *J* = 2.5 Hz, 3′-H), 2.50 (3H, s, CH_3_); ^13^C NMR (150 MHz, DMSO-*d*_6_) *δ* (ppm): 202.75 (C = O), 164.89 (4′-C–OH), 164.21 (2′-C–OH), 133.75 (6′-CH), 112.87 (1′-C), 108.13 (5′-CH), 102.30 (3′-CH), 26.38 (CH_3_). The NMR and mass spectrometry data were in agreement with published structural assignments^37^.

#### General synthesis methods of Q8, Q16 and Q20


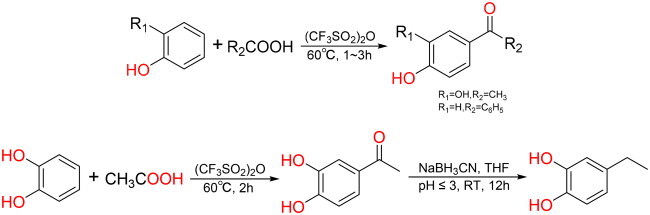


Q16: In a 10 ml round bottom flask, benzoic acid (1 mmol, 122 mg), (CF_3_SO_2_)_2_O (1.1 mmol, 180 μL) and phenol (1 mmol, 94 mg) were added, the reaction were carried out under conditions analogous to those employed for Q14 and Q19. White powder, yield: 17.07%; HR-EIS [M − H]^−^ *m*/*z*: 197.0685; ^1^H NMR (600 MHz, DMSO-*d*_6_) *δ* (ppm): 10.44 (1H, s, 4-OH), 7.65 (4H, d, *J* = 8.5 Hz, 2,6,2′,6′-overlap), 7.62 (1H, t, *J* = 7.4 Hz, 4′-H), 7.52 (2H, t, *J* = 7.7 Hz, 3′,5′–2 × H), 6.89 (2H, d, *J* = 8.8 Hz, 3,5–2 × H)；^13^C NMR (150 MHz, DMSO-*d*_6_) *δ* (ppm): 194.35(C = O), 162.00 (4-C–OH), 138.10 (1′-C), 132.53 (2,6–2 × CH), 131.84 (1-C), 129.14 (2′,6′-2 × CH), 128.40 (3′,5′-2 × CH), 127.90 (4′-CH), 115.27 (3,5–2 × CH). The NMR and mass spectrometry data were in agreement with published structural assignments^38^.

Q20: In a 10 ml round bottom flask, acetic acid (1 mmol, 57 μL), (CF_3_SO_2_)_2_O (1.1 mmol, 180 μL) and catechol (1 mmol, 110 mg) were added, the reaction were carried out under conditions analogous to those employed for Q14 and Q19. Pale yellow solid, yield: 52.40%; HR-EIS [M − H]^−^ *m*/*z*: 151.0394；^1^H NMR (600 MHz, DMSO-*d*_6_) *δ* (ppm): 9.77 (1H, s, 3′-OH), 9.34 (1H, s, 4′-OH), 7.33 (1H, dd, *J* = 8.2, 1.4 Hz, 6′-H), 7.32 (1H, d, *J* = 1.4 Hz, 2′-H), 6.79 (1H, d, *J* = 8.2 Hz, 5′-H), 2.42 (3H, s, CH_3_)；^13^C NMR (150 MHz, DMSO-*d*_6_) *δ* (ppm): 196.13 (C = O), 150.63 (4′-C–OH), 145.13 (3′-C–OH), 128.97 (1′-C), 121.67 (6′-CH), 115.02 (5′-CH), 114.92 (2′-CH), 26.21 (CH_3_). The NMR and mass spectrometry data were in agreement with published structural assignments^39^.

Q8: On the basis of the synthesis of Q20, the next reduction reaction was carried out. In a 25 ml round-bottom flask, 3,4-dihydroxyacetophenone (2 mmol, 304.3 mg), sodium cyanoborohydride (6 mmol, 377.04 mg), about 1 mg methyl orange and 5 ml tetrahydrofuran were added. The solution was mixed and stirred at room temperature and 2 mol/l hydrochloric acid was added drop by drop to keep the solution in red state (pH ≤ 3). Then the solution was magnetically stirred at room temperature for 12 h and the reaction was detected by TLC^40^. At the end of the reaction, 10 ml distilled water was added to quench the reaction system. Then the reaction solution was repeatedly extracted with ethyl acetate, and the ethyl acetate extract was concentrated under reduced pressure. Finally, the product was purified by normal-phase silica gel column chromatography. Brown oil, yield: 96.76%; HR-EIS [M − H]^−^ *m*/*z*: 137.0607; ^1^H NMR (600 MHz, DMSO-*d*_6_) *δ* (ppm): 8.05 (1H, s, 2-OH), 7.72 (1H, s, 1-OH), 6.60 (1H, d, *J* = 7.9 Hz, 6-H), 6.55 (1H, s, 3-H), 6.41 (1H, d, *J* = 8.0 Hz, 5-H), 2.40 (2H, q, *J* = 7.7 Hz, 1′-CH_2_), 1.08 (3H, t, *J* = 7.6 Hz, 2′-CH_3_); ^13^C NMR (150 MHz, DMSO-*d*_6_) *δ* (ppm): 144.97 (2-C–OH), 143.02 (1-C–OH), 134.57 (4-CH), 118.16 (5-CH), 115.39 (6-CH), 115.12 (3-CH), 27.49 (1′-CH_2_), 15.92 (2′-CH_3_). The NMR and mass spectrometry data were in agreement with published structural assignments^41^.

#### General synthesis methods of Q15


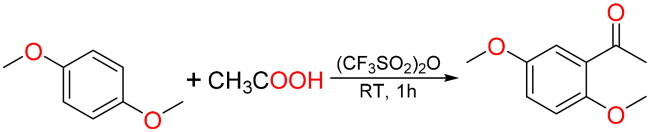


In 10 ml round bottom flask, acetic acid (1 mmol, 57 μL) and (CF_3_SO_2_)_2_O (1.1 mmol, 180 μL) were added, mixed and stirred for 1 min at room temperature, and *p*-dimethoxybenzene (1 mmol, 138 mg) was added. The reaction was stirred for 1 h at room temperature and detected by TLC. After the reaction was completed, 5 ml 10% NaHCO_3_ was slowly added, ethyl acetate was extracted multiple times, the ethyl acetate extract was concentrated under reduced pressure, and the product was purified by normal-phase silica gel column chromatography with ethyl acetate and petroleum ether. Colourless and transparent oil, yield: 58.33%; HR-EIS [M + H]^+^ *m*/*z*: 181.0387; ^1^H NMR (600 MHz, DMSO-*d*_6_) *δ* (ppm): 7.11 (2H, d, *J* = 2.6 Hz, 4′,6′-H), 7.09 (1H, dd, *J* = 2.6, 1.1 Hz, 3′-H), 3.83 (3H, s, 2′-OCH_3_), 3.71 (3H, s, 5′-OCH_3_), 2.51 (3H, s, CH_3_)；^13^C NMR (150 MHz, DMSO-*d*_6_) *δ* (ppm): 198.54 (C = O), 152.84 (2′-C), 152.80 (5′-C), 128.16 (1′-C), 119.60 (4′-CH), 114.08 (3′-CH), 113.42 (6′-CH), 56.26 (2′-OCH_3_), 55.51 (5′-OCH_3_), 31.57 (CH_3_). The NMR and mass spectrometry data were in agreement with published structural assignments^42^.

### Enzyme inhibitory assay

The experiment of mushroom tyrosinase was referred to the article^43^ and modified. In brief, in a 96-well plate, the samples (100 μL DMSO dissolved, PBS buffer (pH = 6.8) diluted to 50 μM) were mixed with mushroom tyrosinase (final concentration of 25 U/mL), and L-Dopa (PBS buffer diluted to 10 mM) was added to start the reaction. Three repeat holes were set, and blank control and kojic acid positive control (100 μL DMSO dissolved, PBS buffer diluted to 50 μM) without compounds were set. After incubation at room temperature for 20 min, the OD value was measured by microplate reader, and the detection wavelength is 490 nm. Finally, the inhibition rate of tyrosinase activity was calculated, values are expressed as the mean ± SD (*n* = 3).

(1) $$\text{Inhibition rate }(\text{\%})\text{ = }1\text{ ‐ }\frac{\text{ Sample OD }}{\text{Experimental control OD}}\;\times \text{ 100\% }$$

### Molecular docking

The binding of Q1-Q12 to mushroom tyrosinase was evaluated by UCSF Chimaera software. The crystal structure of the enzyme (PDB ID: 2Y9X) was from the RCSB protein database (https://www.rcsb.org). The D-chain of mushroom tyrosinase was selected as the docking receptor. After pre-treatment of the receptor and ligand, all molecules were docked by AutoDock Vina program of UCSF Chimaera software. Molecular docking centre X: 21, Y: 3 and Z: −92, size X: 18, Y: 15 and Z: 19. The molecular docking outcomes were subsequently processed through Discovery Studio 2019 client for visualisation and analysis, yielding comprehensive 2D ligand-protein interaction diagrams.

### Zebrafish anti-melanogenic active assay

The whole process of zebrafish whitening efficacy experiments was determined by Guangzhou Hunter Biotechnology Co., Ltd.

Source of animals: Hangzhou Hunter Biotechnology Co., Ltd.

Licence No.: SCXK (Zhejiang) 2022–0003

Justification for model animal used, number of animals, strain, age: zebrafish is transparent in the early stage of development, and melanin begins to grow from the retinal epithelium at 24h of embryonic development. Pigment cells originate from a group of cells differentiated from the dorsal ectoderm-neural crest cells, and then proliferate, migrate, and differentiate into pigment mother cells. Intervention in the process of melanin formation can inhibit the melanogenesis. Wild-type AB strain zebrafish was selected as the experimental object. Zebrafish age: 6 h after fertilisation (6 hpf). Each group of experimental sample size: 15 tails (*N* = 10).

Information on husbandry (housing, feeding, access to water, dark/light cycles etc.): temperature 28.5 ± 0.5 °C, the light/dark cycle was maintained at 14:10 h, the aquatic environment required a conductivity range of 450–650 μS/cm, pH 6.8–7.5.

Information on comfort following procedures (mode of analgesia and anaesthesia and steps taken to minimise suffering): No surgical intervention was required for this assay. Following standard experimental protocols, subjects were to be transferred to water for spontaneous revival.

Endpoints and method of euthanasia: the end point of the experiment was 51 h after fertilisation, that is, 51 hpf, frozen to death.

The general experimental steps were as follows:

1. Six wild-type AB strain zebrafish at 6 h post-fertilisation (hpf) were randomly selected and distributed into a 12-well plate (6 fish per well).
2. The tested compounds were dissolved in aqueous solution and administered to the experimental groups, while a normal control group was established (1 mL/well). The normal control group referred to replacing the added sample concentrations with an equal volume of water during the experiments, the remaining steps were consistent with the operation of the sample groups.
3. The plate was incubated at 28 °C under light-protected condition for 45 h.
4. From each experimental group, three zebrafish were randomly chosen and positioned under an anatomical microscope for imaging. Data were acquired and analysed using advanced image processing software to quantify melanin signal intensity (S) in the head region. Based on the Equation (2), the whitening efficacy of the sample was calculated.
  (2) $$\text{Whitening efficacy }(\text{\%})\text{ = }\frac{\;S1\text{ ‐ S2 }}{S1}\;\times \text{ 100\%}$$

Note: S1 is the normal control group, S2 is the sample group.

Judgement basis: statistical analysis *p* < 0.05, efficacy value ≥ 20%, judged to be effective.

## Results and discussion

### Enzyme inhibitory assay

**Figure 1.**
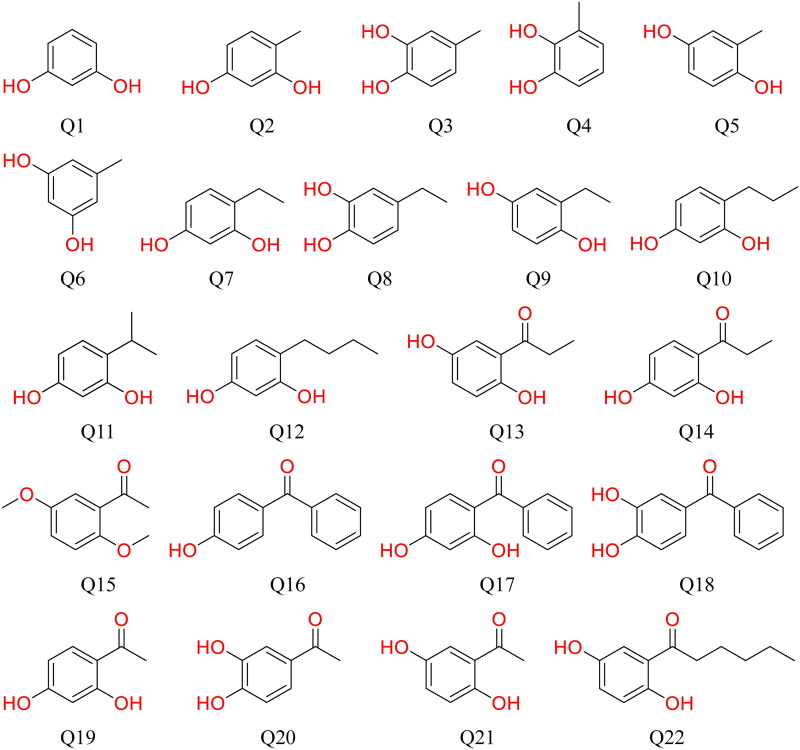
The chemical structure of 22 compounds evaluated for anti-melanogenic activity.

In this tyrosinase activity inhibition assay, 22 compounds (Figure 1) were evaluated for *in vitro* inhibitory activity of mushroom tyrosinase, and the results were shown in Table 1. Compared to the positive control kojic acid, Q2, Q7, Q10 and Q12 demonstrated stronger inhibitory effects, while Q8, Q9 and Q11 showed weaker inhibition, the remaining derivatives showed negligible tyrosinase inhibitory effects.

**Table 1.** The inhibition rate of 22 compounds on tyrosinase.

Compound	Inhibition rate(50 μM, mean ± SD, *n* = 3)
Kojic acid	72.18 ± 2.38
Q1	−17.31 ± 2.40
Q2	75.14 ± 0.48
Q3	−15.95 ± 3.06
Q4	−56.15 ± 4.64
Q5	4.19 ± 2.45
Q6	−34.00 ± 9.02
Q7	78.72 ± 1.85
Q8	23.22 ± 5.57
Q9	13.93 ± 3.77
Q10	82.23 ± 1.51
Q11	57.82 ± 0.91
Q12	83.81 ± 0.65
Q13	−24.26 ± 2.08
Q14	−17.56 ± 0.91
Q15	−0.99 ± 1.87
Q16	−25.59 ± 3.29
Q17	−24.33 ± 4.20
Q18	−12.32 ± 2.13
Q19	−12.12 ± 3.25
Q20	7.79 ± 1.85
Q21	−10.29 ± 2.96
Q22	−8.38 ± 4.25

The mushroom tyrosinase assay served only as a preliminary screening test without IC_50_ determination, with our primary findings being based on zebrafish experimental results.

In this assay, 4-methyl-1,3-benzenediol (Q2) exhibited a higher inhibition rate than kojic acid, whereas unsubstituted 1,3-benzenediol showed no inhibitory activity. This suggests that introducing a methyl group at the 4-position of resorcinol enhances hydrophobic interactions with the tyrosinase active pocket, thereby improving binding affinity and inhibitory efficacy against mushroom tyrosinase. Furthermore, activity tests were performed on structural isomers of 4-methyl-1,3-benzenediol, but none demonstrated comparable inhibition (Table 1). The results indicate that 4-methyl-1,3-benzenediol serves as an effective structural motif for tyrosinase inhibition *in vitro*, providing a valuable template for the rational design of potential tyrosinase inhibitors.

Based on these findings, the three ethyl-substituted dihydroxybenzene isomers (ortho-, meta-, and para-) were evaluated. The results demonstrated that both 4-ethylcatechol (Q8) and 2-ethylhydroquinone (Q9) exhibited significantly weaker inhibitory effects compared to 4-ethylresorcinol (Q7) (Table 1). It was concluded from these results that for these three ethyl-substituted dihydroxybenzene isomers, not only the position of the ethyl group on the benzene ring, but also the relative positions of the phenolic hydroxyl groups substantially influenced the inhibitory activity against mushroom tyrosinase.

### Molecular docking

**Figure 2.**
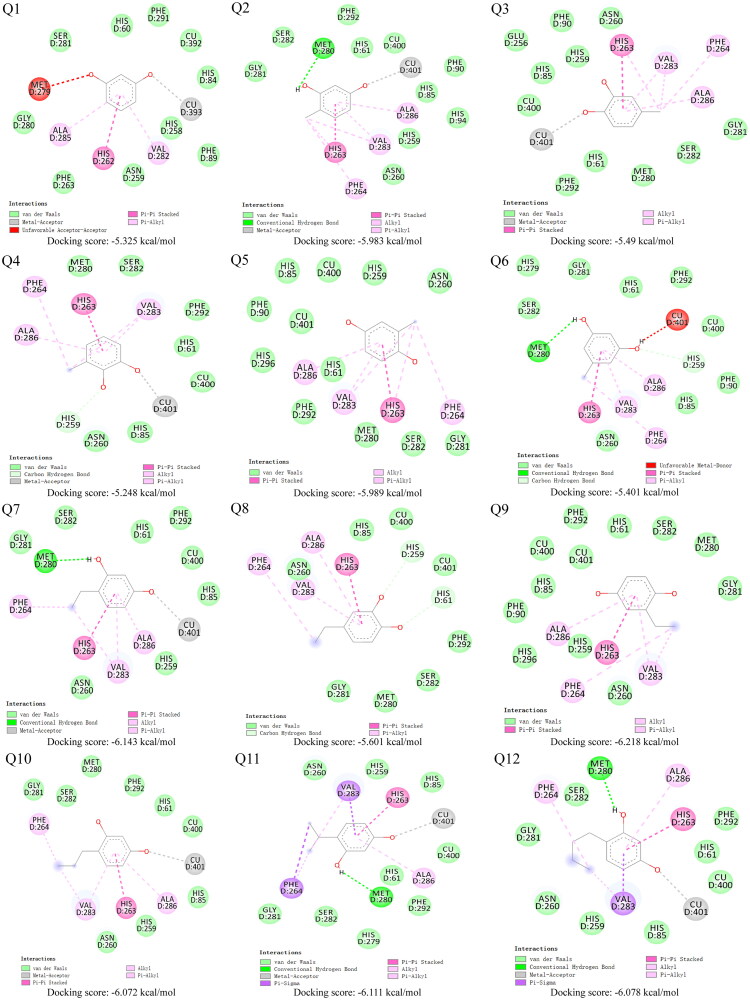
Docking results of compound Q1-Q12 with tyrosinase. Green dotted line represents hydrogen bond, light pink dotted line represents hydrophobic interaction, grey dotted line represents electrostatic interaction, the pink dotted line represents the π–π interaction, the purple dotted line represents the π–σ interaction, the red dotted line indicates unfavourable factors.

Molecular docking studies of Q1-Q12 with mushroom tyrosinase were performed to investigate the influence of structural variations on binding site interactions. All ligands were located in the same binding pocket (Figure 2). The docking results revealed that the isomeric configuration of the phenolic hydroxyl groups predominantly governed inhibitory potency, followed by steric constraints between the ligands and the receptor within the active site. Among the derivatives, 4-alkylresorcinol analogs exhibited the most favourable binding poses, with one phenolic hydroxyl group coordinating to the copper ion and the other forming a hydrogen bond with Met280, while the 4-alkyl chain engaged in hydrophobic interactions with Val283.

Furthermore, analysis of Q2 and Q6 demonstrated that the positional orientation of the methyl substituent on the benzene ring significantly influenced steric hindrance within the active pocket. Methyl substitution at the 4-position proved optimal, facilitating closer proximity to the copper ion and enhancing inhibitory efficacy. Collectively, hydrogen bonding, electrostatic interactions, and hydrophobic effects were critical for the binding of Q2, Q7, Q10, Q11, and Q12 to mushroom tyrosinase. The docking observations correlated well with the enzymatic inhibition data, validating the computational model.

**Figure 3.**
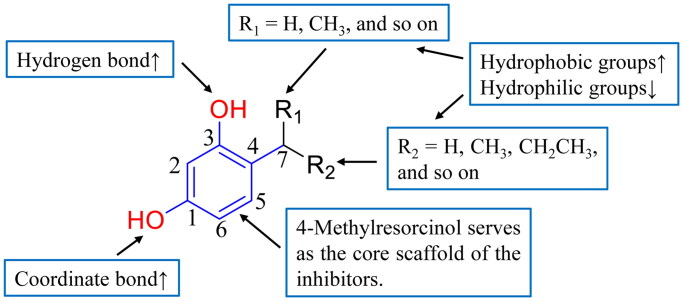
The structure-activity relationship (SAR) of mushroom tyrosinase inhibitors. “↑” indicates enhanced inhibitory effect, “↓” denotes reduced inhibitory effect.

Based on the comprehensive analysis of experimental results and molecular docking studies, the structure-activity relationship (SAR) of mushroom tyrosinase inhibitors was systematically elucidated in this study (Figure 3). Structure-activity relationship analysis revealed that the phenolic hydroxyl group at the 3-position serves as an essential moiety, forming hydrogen bonds with amino acid residues to enhance molecular interactions. The phenolic hydroxyl group at the 1-position was identified as an important functional group that coordinates with copper ions. Furthermore, the 7-position was found to tolerate various substituents, though groups capable of forming intramolecular hydrogen bonds with the 3-position hydroxyl (such as carbonyl groups) should be avoided. Notably, the introduction of hydrophobic substituents at the 7-position significantly enhanced the inhibitory activity of the compounds.

### Zebrafish anti-melanogenic active assay

As tyrosinase inhibition was evaluated in an *in vitro* model, further validation of the anti-melanogenic effects was subsequently performed using a zebrafish model. This approach was adopted because previous studies had reported that certain compounds demonstrating tyrosinase inhibitory activity *in vitro* failed to exhibit efficacy *in vivo*^10^. The observed discrepancy was attributed to nucleotide sequence variations between mushroom tyrosinase and its zebrafish/animal counterparts^44^, suggesting that these compounds might not effectively inhibit animal-derived tyrosinase. Based on these findings, it was postulated that 2,5-dihydroxyacetophenone likely exerted its effects through tyrosinase modulation^32^. Consequently, comprehensive evaluation of all compounds for their inhibitory effects on melanogenesis in zebrafish was conducted to verify their *in vivo* efficacy.

In the zebrafish assay, 2,5-dihydroxyacetophenone isolated from *Ganoderma cochlear* and the positive control SymWhite^®^377 (phenylethyl resorcinol) showed melanin inhibition rates of 82% (*c* = 0.0025%) and 15% (*c* = 0.0005%), respectively. These results indicated that 2,5-dihydroxyacetophenone serves as a potent melanin synthesis inhibitor. Furthermore, no toxic effects were observed during the complete embryonic-to-larval development of zebrafish, confirming the compound had excellent safety profile.

**Figure 4.**
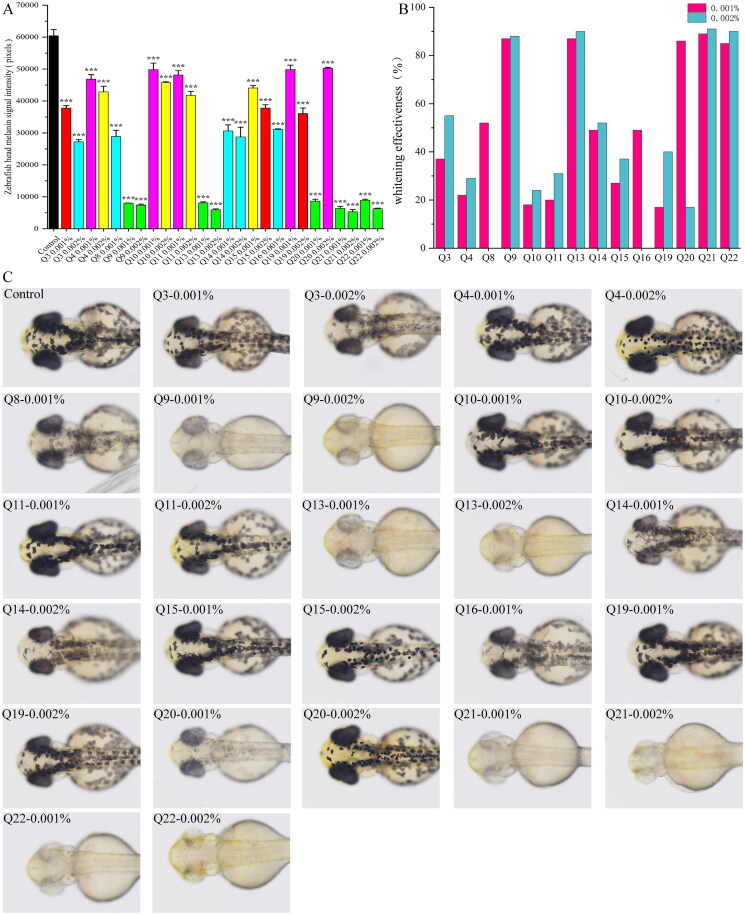
(A) the bar chart of melanin signal intensity in zebrafish head, (B) the whitening efficacies of zebrafish at different concentrations, (C) the results of inhibition of melanin formation in zebrafish by some compounds. Compared with the normal control group, * *p* < 0.05, ** *p* < 0.01, *** *p* < 0.001, statistical analysis *p* < 0.05, efficacy value ≥ 20%, judged to be effective.

**Figure 5.**
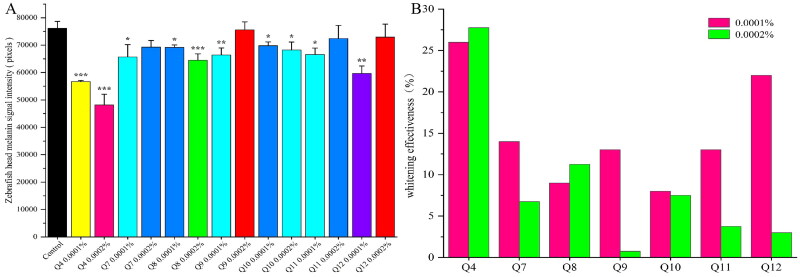
A: the bar chart of melanin signal intensity in zebrafish head, B: the whitening efficacies of zebrafish at different concentrations. Compared with the normal control group, *p < 0.05, **p < 0.01, ***p < 0.001, statistical analysis *p* < 0.05, efficacy value ≥ 20%, judged to be effective.

Based on these findings, 2,5-dihydroxyacetophenone and its structural analogs were further investigated to evaluate their melanogenesis inhibitory effects. Twenty compounds (excluding Q1 and Q6) were tested at different concentrations for zebrafish melanin inhibition (Figure 4). Compared with the normal control group, Q9, Q13, Q21, and Q22 exhibited potent inhibitory effects at both tested concentrations. Q8, Q16, and Q20 showed significant inhibition at 0.001%, but their efficacy was diminished or accompanied by toxicity at 0.002%. Moderate concentration-dependent inhibition was observed for Q3, Q4, Q10, Q11, Q14, Q15, and Q19, with their inhibitory rates increasing proportionally with concentration. However, the remaining compounds induced either lethality or developmental abnormalities in zebrafish at both 0.001% and 0.002%. Due to the observed toxicity, selected compounds were re-evaluated at lower concentrations (*c* = 0.0001% and 0.0002%) (Figure 5). Notably, Q5 still demonstrated 100% lethality in zebrafish even at these reduced concentrations.

The results revealed that the substitution pattern of phenolic hydroxyl groups on the benzene ring exerted the most pronounced influence on melanogenesis inhibition, with hydroquinone derivatives demonstrating optimal inhibitory activity. A significant reduction in efficacy was observed upon methylation of both para-positioned phenolic hydroxyl groups (Figure 4), indicating that the presence of free para-hydroxyl groups is critical for anti-melanogenic activity. Catechol derivatives were found to exhibit significant inhibitory effects on zebrafish melanogenesis. Among these, compound Q20 demonstrated optimal efficacy at a concentration of 0.001%, while its inhibitory activity was observed to decrease dramatically at 0.002%, suggesting that the most effective concentration for Q20 is approximately 0.001%. Regarding resorcinol derivatives, moderate melanogenesis inhibition was observed. The most potent inhibition was displayed by resorcinol derivatives containing an α-carbonyl group on the side chain of the benzene ring. Q16, phenolic derivative, exhibited moderate whitening efficacy in zebrafish (49% melanin inhibition at 0.001%). However, pericardial haemorrhage was observed at 0.002%, the steep toxicity threshold of Q16 (effective dose 0.001% vs toxic dose 0.002%) implied that minor structural modifications might be required to improve its safety profile.

**Figure 6.**
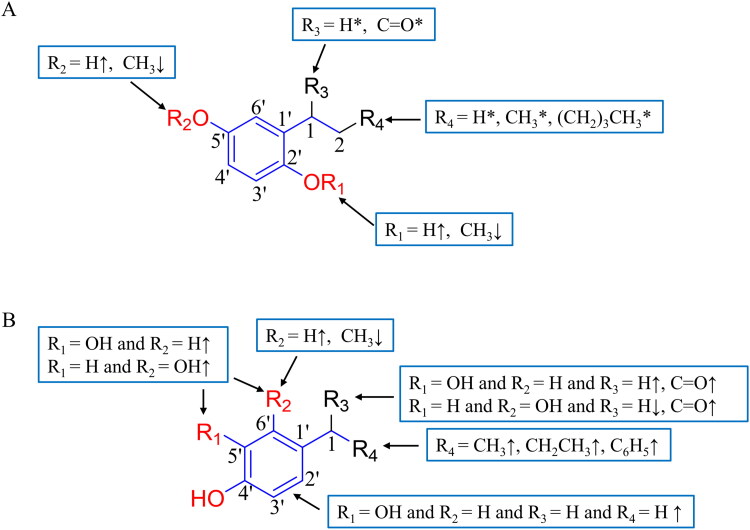
The structure-activity relationship (SAR) of anti-melanogenic compounds. 1: (A) the most potent compounds; (B) moderately to highly effective ones. 2: “↑” indicates enhanced inhibitory effect, “↓” denotes reduced inhibitory effect, “*” represents no significant change in inhibitory activity.

Based on the zebrafish experimental results, the structure-activity relationship (SAR) of anti-melanogenic inhibitors was established (Figure 6). Interestingly, compounds that showed no inhibitory activity against mushroom tyrosinase were found to exhibit potent melanogenesis suppression in zebrafish, whereas those demonstrating strong inhibition in the mushroom tyrosinase assay failed to show significant effects in the zebrafish model. This discrepancy highlights that zebrafish experimental results should serve as the primary standard for evaluating melanin inhibition efficacy, as *in vivo* experiments generally provide more reliable data than *in vitro* assays. Moreover, the zebrafish model, incorporating physiological absorption and metabolism, offers a more comprehensive assessment of anti-melanogenic potential.

**Figure 7.**
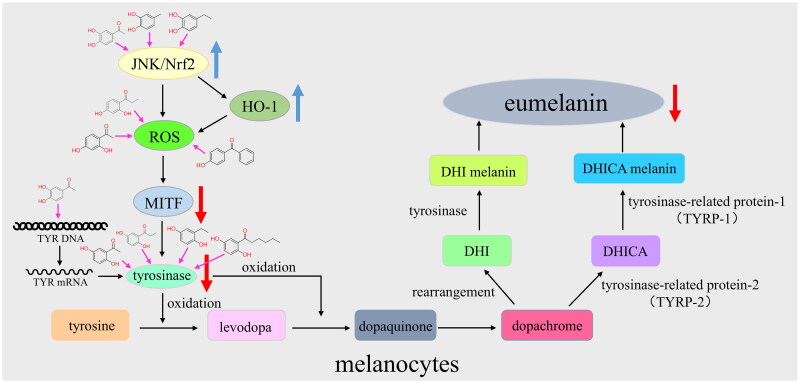
The possible mechanisms of inhibiting melanin synthesis.

The inhibiting mechanism of the target compounds was speculated through comparative structural analysis with reported active molecules^32^^,^^45–50^. In the zebrafish skin-whitening efficacy experiments, the active sites and potential mechanisms of compounds demonstrating superior melanin inhibition were analysed based on the experimental results (Figure 7). Q9, Q13, Q21, and Q22 appear to primarily target tyrosinase activity. Q3, Q4, and Q8 seem to reduce melanin synthesis mainly through antioxidant pathways. Q20 may exhibit a dual mechanism, potentially combining tyrosinase gene downregulation with antioxidant effects. Q10, Q11, Q14, and Q19 might interfere with TRP-2 expression or act through alternative antioxidant mechanisms. Q16 could be predominantly mediated by its antioxidant properties.

## Conclusions

2,5-Dihydroxyacetophenone was demonstrated to effectively inhibit tyrosinase activity and reduce melanin production in both murine and zebrafish models, establishing it as a potent yet low-toxicity natural anti-melanogenic agent.

In this study, a diverse series of structurally modified analogs, based on the 2,5-dihydroxyacetophenone scaffold, were synthesised or commercially procured for comprehensive evaluation. Through systematic enzyme assays and zebrafish testing, fourteen compounds (Q3, Q4, Q8-Q11, Q13-Q16, Q19-Q22) were identified as effective melanogenesis inhibitors. Among these, Q9, Q13, Q20, Q21, and Q22 exhibited the most pronounced inhibitory effects, providing a robust theoretical foundation for future development of depigmenting agents.

## Data Availability

The data that support the findings of this study are available from the corresponding author, Mr. Qiu, upon reasonable request.

## Health and safety

We confirm that all mandatory laboratory health and safety procedures have been complied within the course of conducting any experimental work reported in the paper.
